# *Ophiorrhiza monsvictoriae* (Rubiaceae, Rubioideae), a new species from Myanmar

**DOI:** 10.3897/phytokeys.138.38966

**Published:** 2020-01-10

**Authors:** Shi-Shun Zhou, Ren Li, Rui-Chang Quan, Law Shine, Lin-Dong Duan

**Affiliations:** 1 Center for Integrative Conservation, Xishuangbanna Tropical Botanical Garden, Chinese Academy of Sciences, China Xishuangbanna Tropical Botanical Garden, Chinese Academy of Sciences Xishuangbanna China; 2 Southeast Asia Biodiversity Research Institute, Chinese Academy of Sciences, Yezin, Nay Pyi Taw 05282, Myanmar Southeast Asia Biodiversity Research Institute, Chinese Academy of Sciences Nay Pyi Taw Myanmar; 3 Natma Taung National Park, Natural and Wildlife Conservation Division, Forest Department, Myanmar Natma Taung National Park, Natural and Wildlife Conservation Division, Forest Department Kanpetlet Myanmar; 4 Shaoyang University, Shaoyang 422004, Hunan, China Shaoyang University Shaoyang China

**Keywords:** NatmaTaung (Mt. Victoria) National Park, taxonomy, new taxon

## Abstract

A new species, *Ophiorrhiza monsvictoriae* S.S.Zhou & L.D.Duan, discovered at Natma Taung (Mt.Victoria) National Park, Chin State, Myanmar, is described and illustrated. The new species is morphologically similar to *O. dulongensis*, but differs from the latter by its stipules broadly triangular, 2–4 mm long, inflorescence axillary, 1-flowered, bracts lanceolate to subulate, 1–2 mm long, puberulous, calyx pilose, 1–2 mm long, lobes and tube equal length, corolla narrowly funnelform, 15 mm long, puberulous outside.

## Introduction

The genus *Ophiorriza* Linnaeus belongs to the tribe *Ophiorrhizeae* in the subfamily Rubioideae (Bremer and Manen 2000; Duan et al. 2019). It is a notably species-rich, taxonomically complicated genus consisting of 318 species, five varieties and one subspecies and found in wet tropical forests of South-East Asia and extending to Australia, New Guinea and the Pacific islands (Darwin 1976; Chen and Taylor 2011). Approximately 18 species of this genus have been recorded from Myanmar (Kress et al. 2003).

The Natma Taung (Mt. Victoria) National Park is located in the south-western part of Myanmar. Mount Victoria is the highest mountain in this region and has been regarded as an ecological refugium, offering a temperate zone that is absent from neighboring regions (Tanaka et al. 2010a). Belonging to the world biodiversity research hotspot areas, it is estimated that there are about 2500 vascular plant species on Mt. Victoria and a number of endemic, relict and new species have been found in this area (Cowley 1982; Tanaka et al. 2010b; Zhou et al. 2018; Ding et al. 2019). We carried out field expeditions in this area since 2016, sponsored by the Xishuangbanna Tropical Botanical Garden, CAS, in cooperation with the Forest Department, Ministry of Natural Resources and Environmental Conservation, Myanmar. A new species of *Ophiorrhiza* was discovered and is described below. The new species belongs to Ophiorrhiza section Proliferae (Lindl.) Pfitzer and Kraenzlin.

## Materials and methods

A morphological description (Stearn 1983) of the new species was prepared from living plants and five dried herbarium specimens (HITBC: herbaria of Xishuangbanna Tropical Botanical Garden, the Chinese Academy of Science). Measurements were made using a vernier caliper. Herbarium and fresh specimens of *Ophiorrhiza dulongensis* (KUN: herbaria of Kunming Institute of Botany, the Chinese Academy of Science) (Lo 1990) were examined. The conservation status of the new species was evaluated based on the International Union for Conservation of Nature criteria C (Small population size and decline). We just observed the number of mature individuals in the subpopulation and criteria of C2a [i] is used to evaluate the threatened status (vulnerable) (IUCN Standards and Petitions Subcommittee 2017).

## Taxonomy

### Ophiorrhiza monsvictoriae

Taxon classificationPlantaeGentianalesRubiaceae

S.S.Zhou & L.D.Duan sp. nov.

C7B4DA65-2743-5B81-BD0A-23431AE2DE95

urn:lsid:ipni.org:names:77204218-1

Fig. 1

#### Diagnosis.

*Ophiorrhiza monsvictoriae* is similar to *Ophiorrhiza dulongensis* H. S. Lo (1990: 27), but differs from it by the principal veins raised on both sides of leaf, stipules broadly triangular, 2–4 mm long, inflorescences axillary, 1-flowered, peduncles puberulous, bracts lanceolate to subulate, 1–2 mm long, puberulous, calyx pilose, 1–2 mm long, lobes and tube equal in length, and corolla narrowly funnelform, 15 mm long, puberulous outside, see Table 1.

**Table 1.** Diagnostic morphological characters of *Ophiorrhiza dulongensis* and *O. monsvictoriae*.

Characters	*O. dulongensis*	*O. monsvictoriae*
Leaf	principal veins flat on both sides of leaf blade; stipules subulate, 4–6 mm long	principal veins raised on both sides of leaf blade; stipules broadly triangular, 2–4 mm long
Inflorescence	inflorescence fasciculate, 3- or 4-flowered; peduncle glabrous; bracts linear, ca. 1 mm long, glabrous	inflorescence axillary, 1-flowered; peduncle puberulous; bracts lanceolate to subulate, 1–2mm long, puberulous
Flower	calyx puberulent to glabrescent, 3–4 mm long; lobes slightly longer than calyx tube; corolla funnelform, 11 mm long, glabrate outside	calyx pilose,1–2 mm long, lobes and tube equal in length; corolla narrowly funnelform, 15 mm long, puberulous outside

#### Type.

MYANMAR. Chin State. Natma Taung (Mt. Victoria) National Park, under evergreen broad-leaved forest in tropical mountainous areas forest, 2500–2600 m, 18 July 2018, Shi Shun-Zhou 15305 (holotype: RAF!; isotype: HITBC!, Herb. Bar. Code No. 169316).

#### Description.

Herbs, creeping to weakly ascending, 15–30 cm tall; stems drying purplish brown, puberulous. Petioles 0.5–1 cm long, puberulous; leaf blade drying membranous to papery, adaxially green, abaxially grayish-green, broadly ovate or elliptic, 2–4 × 1–2.5 cm, adaxially scattered puberulous, abaxially moderately puberulous along principal veins, base obtuse, apex acute; secondary veins 4–5 pairs; stipules broadly triangular, 2–4 mm long, glabrous. Inflorescences axillary, 1-flowered; peduncle puberulous, 6–8 mm long; pedicel ca. 2 mm long, puberulous; macrostylous: bracts lanceolate to subulate, 1–2mm, puberulous. Calyx pilose, 1–2 mm; hypanthium 5-ribbed; lobes lanceolate; lobes and tube equal in length. Corolla white, 15 mm long, narrowly funnelform, puberulous outside, pilose inside; tube densely villous in throat; lobes triangular-lanceolate, 3–3.5 mm long, dorsally ribbed at least in bud. Stamens reaching the tube throat; anthers linear; style reaching the tube mouth; Capsules obcordate, ca. 2 × 4.5 mm.

**Figure 1.**
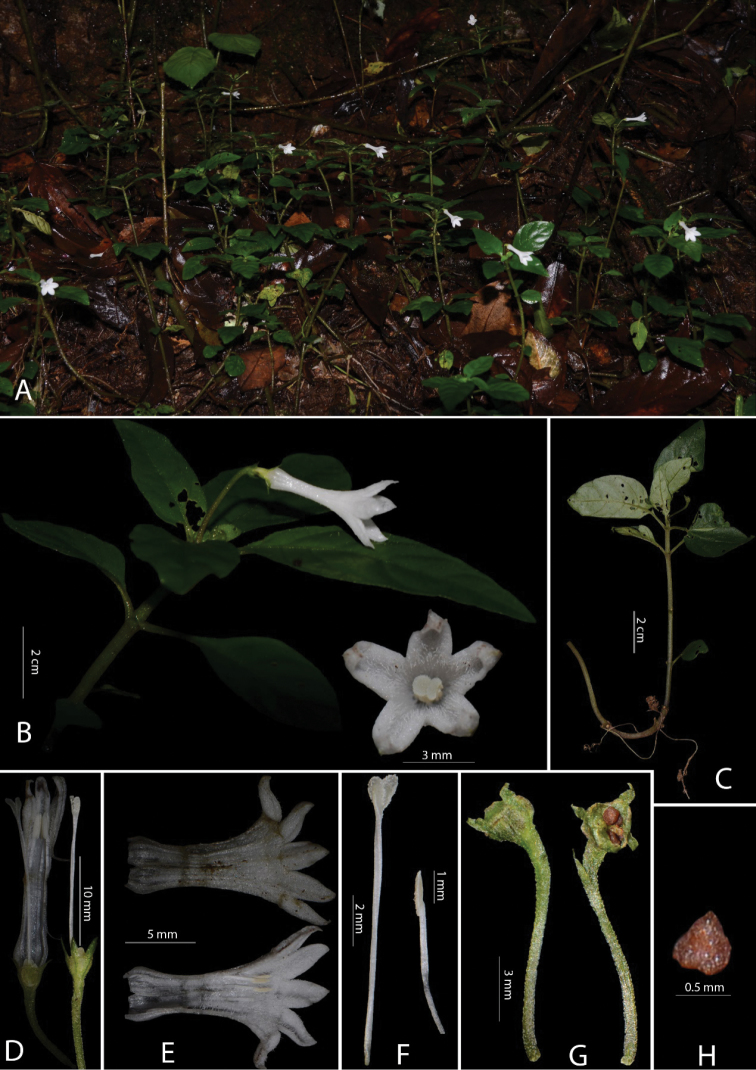
*Ophiorrhiza monsvictoriae* S.S.Zhou & L.D.Duan, sp. nov. **A** Habitat **B** front view of flower and Inflorescence **C** infructescence **D** opened corolla of long styled flower **E** corolla inside and outside **F** stamen and style **G** fruit **H** seed.

#### Etymology.

The new species was named after Mountain Victoria, Natma Taung National Park, Chin State, southwestern Myanmar, where it was discovered in a vast area of mountain forest.

#### Phenology.

Flowering in July to August, fruits in August to September.

#### Distribution and habitat.

*Ophiorrhiza monsvictoriae* is only known from the type locality. It is a terrestrial plant that grows in the floating soil on the stone in subtropical evergreen broad-leaved forest, which is dominated by *Lithocarpus xylocarpus* (Kurz) Markg. (Fagaceae).

#### Conservation status.

*Ophiorrhiza monsvictoriae* was collected on Victoria Mountain, Natma Taung National Park, Chin State, South-western Myanmar. However, only one population, consisting of approximately100 individuals, has been discovered so far in the National Park. Other populations may be found with further investigation because the area is legally protected under by the government of Myanmar.

#### Critical note.

The new species most resembles *Ophiorrhiza dulongensis*. Detailed morphological differences between the two species are given in Table 1.

## Supplementary Material

XML Treatment for Ophiorrhiza monsvictoriae
